# A 2–6 GHz Ultra-Wideband Shared-Aperture Antenna Array for 5G Multi-Band Base Station

**DOI:** 10.3390/mi17040485

**Published:** 2026-04-16

**Authors:** Lingang Yang, Junkai He, Yuqing Gao, Yue Wang, Jun Wang

**Affiliations:** 1 Power China Huadong Engineering Corporation Limited, Hangzhou 311122, China; yang_lg@hdec.com (L.Y.); gao_yq@hdec.com (Y.G.); 2 School of Information and Control Engineering, China University of Mining and Technology, Xuzhou 221116, China; ts25060141p31@cumt.edu.cn

**Keywords:** 5G base station antenna, shared-aperture base station antenna, broadband, multi-band

## Abstract

This paper proposes a non-overlapping planar cross-arranged ultra-wideband shared-aperture base station antenna array targeting the 2 to 6 GHz application bandwidth. The low-frequency module (double-layer parasitic coupling) and the high-frequency module (chamfered slotted patch) are independently designed, and metal baffles are introduced around the antenna elements to reshape the boundary conditions and physically block the electromagnetic coupling paths. Both simulation and experimental results demonstrate that the fabricated prototype successfully exceeds the targeted 2–6 GHz spectrum, achieving an actual continuous coverage from 1.84 to 6.3 GHz. Specifically, the antenna achieves a gain higher than 5.9 dBi in the measured low-frequency band (1.84–3.72 GHz) and higher than 6.1 dBi in the high-frequency band (3.63–6.3 GHz), with a voltage standing wave ratio (VSWR) below 2 across the entire band. The metal baffles successfully correct the high-frequency radiation pattern distortion and ensure stable directional radiation over the full operating bandwidth. This design provides an efficient, robust, and manufacturable solution for 5G offshore wind power multi-band base station antennas.

## 1. Introduction

With the wide-range coverage of 5G networks and the wide commercial use of 3rd Generation Partnership Project (3GPP) Release 16/17 standards [1], the Sub-6 GHz frequency band, due to its advantages such as wide coverage range, strong penetration ability, and mature technology, is widely applied to network communications in densely populated cities and towns [2]. In order to realize the smooth transition of multi-generational radio access networks and the collaborative deployment of co-site base stations, modern base stations put strict requirements on the ultra-wideband of antennas and the high integration degree of different frequency bands [3]. Specifically, the front-end Radio Frequency (RF) system not only needs to be compatible with traditional 3G/4G frequency bands (such as FDD-LTE Band 1/3/7 and TDD-LTE Band 38/40/41), but also must cover the key working frequency bands of 5G New Radio (NR) (such as 3.3–4.2 GHz of n77/n78, and 4.4–5.0 GHz of n79), and also take into account the license-exempt frequency bands such as Citizens Broadband Radio Service (CBRS, 3.55–3.7 GHz) and 5 GHz Licensed Assisted Access (LAA/Wi-Fi) [4]. Therefore, developing base station antennas that can continuously cover the targeted 2–6 GHz ultra-wideband can effectively solve the problem that base station site resources are limited and realize the integrated and efficient integration of multi-band signals.

In order to integrate antennas of multiple frequency bands within the limited space on the base station tower, the shared-aperture arrangement method has become a current research hotspot. The more widely applied shared-aperture designs include nested type, side-by-side type, stacked type and cross type. The nested type [4] structure, by embedding the high-frequency unit inside the low-frequency unit or in the gaps, thereby achieves the highest space utilization rate, but this structure has the high- and low-frequency units relatively close to each other, and the coupling between units is relatively serious. The side-by-side type [5] is to interleave the high- and low-frequency antenna units within the same plane, without nesting or stacking each other. This structural characteristic leads to its coupling between units being relatively small, the isolation being good, but relative to it the array density is low, and the occupied lateral plane is large. The stacked type [6] structure vertically stacks the high- and low-frequency units at different height layers; usually, the low frequency is below, the high frequency is above, its longitudinally integrated structure makes the high- and low-frequency units relatively independent, and the performance is easy to ensure. But the antenna layer-by-layer stacking will also lead to the overall machine cross-section thickness being large. The cross type [7] structure cross-arranges the high- and low-frequency units in the same plane, placing the low-frequency antenna in the middle and arranging the high-frequency antennas around. Such a structure is simple, the beam symmetry is good, and it is conducive to dual polarization, but the physical overlap region between the high- and low-frequency units easily produces coupling, leading to radiation pattern distortion.

When the distance between the high- and low-frequency antenna units is too close, the radiation of the two will interfere with each other [8]. In order to suppress the scattering interference between the high- and low-frequency antenna units, researchers have proposed a variety of decoupling techniques, such as loading U-shaped slots on the low-frequency antenna [9], loading choke structures [10] and integrating Frequency Selective Surface (FSS) structures [11], etc. In order to suppress the common-mode interference between units, researchers have proposed the path extension method [12], the method of adding ferrite on the coaxial feed of the high-frequency antenna [13], and the method of loading an Inductor–Capacitor (LC) circuit between the high-frequency coaxial feedline and the ground plane [14], etc.

In this paper, a non-overlapping planar cross-arrangement structure is adopted to design ultra-wideband shared-aperture base station antenna array with an operating frequency band of 1.84–6.3 GHz while maintaining aperture compactness. In order to solve the coupling problem between units, metal baffles are added around the low-frequency and high-frequency units. The metal baffles physically reshape the boundary conditions and effectively cut off the electromagnetic wave coupling path between the high- and low-frequency units. The experimental and simulation results show that the antenna maintains good impedance matching in both the low-frequency band (1.84–3.72 GHz) and the high-frequency band (3.63–6.3 GHz), the voltage standing wave ratio is below 2 within the operating band, and port isolation higher than 35 dB is realized in the overlapping band of 3.63–3.72 GHz. In addition, the antenna after introducing the baffles shows stable radiation patterns and higher isolation over the entire operating frequency band, providing an efficient, robust, and easy-to-manufacture solution for the 5G offshore wind power multi-band base station antennas.

## 2. Antenna Design and Electromagnetic Simulation

The proposed antenna adopts a shared-aperture collaborative strategy of independent design for the high- and low-frequency bands, which splits the overall system into a low-frequency module of 1.84–3.72 GHz and a high-frequency module of 3.63–6.3GHz. The design optimizes the respective resonant units and feed networks by modules, and arranges them in a non-overlapping cross arrangement in the plane, while maximizing the use of space and ensuring the overall stable radiation efficiency.

### 2.1. High-Band Antenna

In order to realize wideband performance, the patch corners and opening slots are conducted to improve its electromagnetic characteristics. By chamfering the patch corners to remove the charge accumulation at the right-angle corners, the current is forced to deviate from the original transmission path; this change in boundary greatly lengthens the actual transmission path of the current, increases the equivalent electrical length without enlarging the patch size, and moves the resonance point toward low frequency. Etching an annular slot inside the radiating patch creates a more tortuous path for the current. The resulting gaps introduce additional equivalent capacitance and inductance, which excite new high-frequency resonant modes. Finally, the superposition of these multiple resonant peaks achieves continuous ultra-wideband coverage.

**Figure 1:**
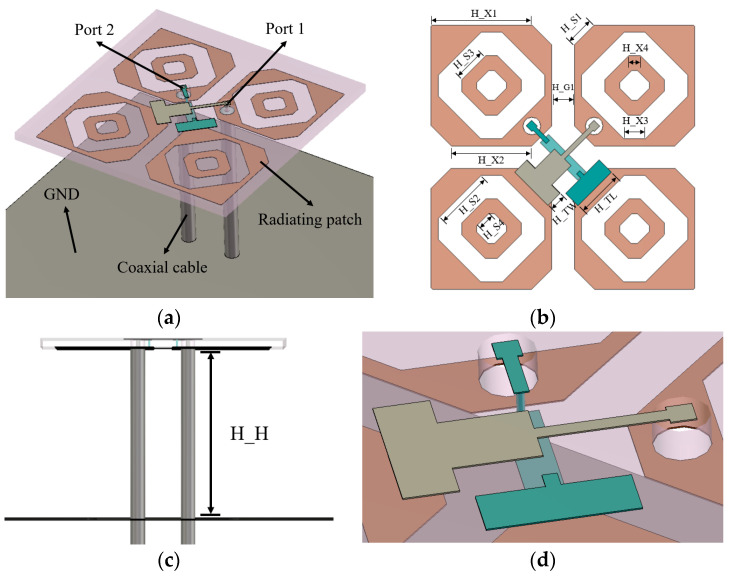
Structure and dimensions of the high-frequency antenna: (**a**) overall structure; (**b**) top view with detailed dimensional parameters; (**c**) side view; (**d**) T-shaped feeding probe.

The proposed antenna element is shown in Figure 1. The radiating patch consists of four symmetrical slotted chamfered patches, forming a ±45° orthogonal dual-polarization layout. The feeding probes and the radiating patches are etched on the two side surfaces of an FR4 dielectric substrate with a thickness of 0.8 mm (relative permittivity ε_r_ = 4.3, loss tangent tan δ = 0.025). To prevent short-circuiting at the intersection of the two T-shaped feeding probes, a cross-layer routing strategy is introduced. A 50 Ω coaxial line feeds the system. Its outer conductor connects directly to the bottom radiating patch, while the inner conductor passes through the substrate to connect to the top T-shaped probe. Ultimately, the capacitive coupling provided by this T-shaped probe cancels the high-frequency inductive reactance, achieving broadband matching. The detailed dimensional parameters of the high-frequency antenna are shown in Table 1.

**Figure 2:**
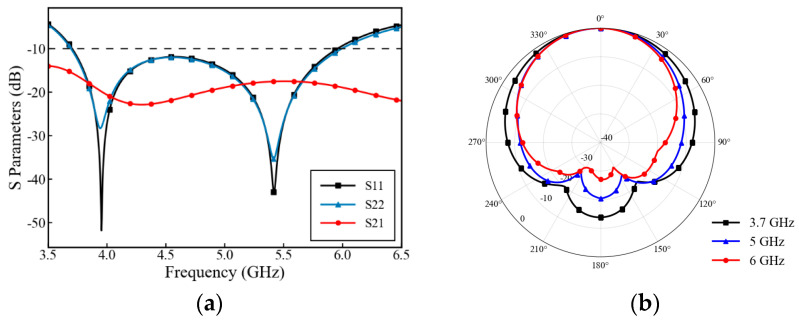
Simulated performance of the high-frequency antenna: (**a**) S-parameters; (**b**) normalized main-polarization radiation patterns at 3.7 GHz, 5 GHz, and 6 GHz.

Figure 2a shows the S-parameter curves of the high-frequency antenna element. The operating frequency band is 3.68–6 GHz, exhibiting clear dual-resonance pole characteristics (located near 3.9 GHz and 5.4 GHz, respectively), with a relative bandwidth of 47.9%. With port isolation exceeding 15 dB, the operating band covers the 5G band n79 (4.4–5.0 GHz), the 4G LTE evolved Band 46 (5.15–5.925 GHz), and the C-V2X/ITS vehicular network band (5.85–5.925 GHz).

Ports 1 and 2 exhibit different impedance matching due to their distinct feeding probe structures. Port 1 adopts a same-layer probe structure, with a short feeding path and relatively stable parasitic inductance. Port 2 features a three-dimensional cross-layer probe structure that introduces additional metal vias and backside microstrip lines. The metal via itself can be equivalent to a series inductance L_p_, and the top-layer probe and the bottom-layer routing form an additional equivalent capacitance C_c_. The introduction of these parasitic parameters is reflected in the differences in the resonance frequency and matching depth. Figure 2b shows the normalized radiation patterns of the high-frequency antenna element at 3.7 GHz, 5 GHz, and 6 GHz. It can be seen that the main radiation directions are all concentrated near 0°, exhibiting good wide-beam directional radiation characteristics.

### 2.2. Low-Band Antenna

To realize the low-frequency resonance characteristics, a double-layer stacked parasitic coupling antenna is proposed in this section. This design retains the traditional method of increasing physical size through chamfering, while simultaneously introducing a multi-mode electromagnetic coupling mechanism. By utilizing the parasitic layer to excite and superimpose additional resonance points, the antenna’s operating bandwidth is effectively broadened.

**Figure 3:**
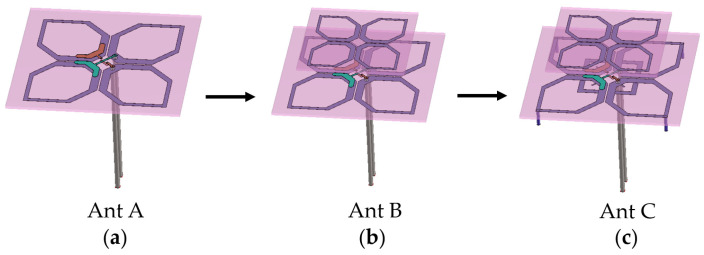
Structural evolution of the proposed antenna: (**a**) initial reference antenna; (**b**) first iteration with a stacked parasitic structure layer; (**c**) final optimized antenna with corner-loaded metal pillars and middle short metal paths.

**Figure 4:**
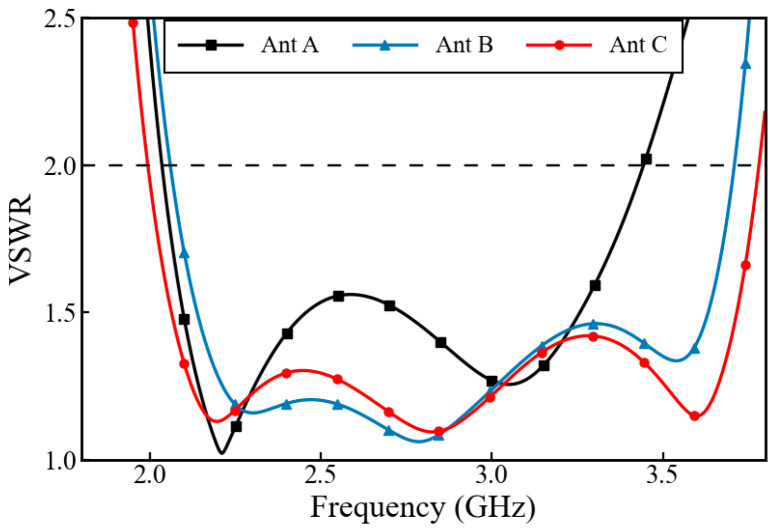
VSWR comparison of the low-frequency antenna iterations.

Figure 3 shows the three main evolution stages of the low-frequency antenna element structure. As shown in Figure 3a, the initial low-frequency radiating element adopts a traditional dipole design. The main radiator consists of four metal ring arms arranged in ±45°, etched on the bottom of an FR-4 dielectric substrate with a thickness of 0.8 mm (relative permittivity ε_r_ = 4.3, loss tangent tan δ = 0.025). The feeding method is the same as that of the high-frequency antenna, both adopting a 50 Ω coaxial line, and the inner conductor is soldered to the Y-shaped probe for feeding. It can be seen from the VSWR curve in Figure 4 that although the initial antenna A (Ant A) has resonances near 2.2 GHz and 3.0 GHz, its high-frequency end matching is poor. After 3.4 GHz, the VSWR deteriorates rapidly and exceeds 2.0, making it difficult to meet the coverage requirements of ultra-wideband. By scaling down the initial radiating element and separating it with an air gap, antenna B (Ant B) is created as a stacked parasitic structure. This vertical arrangement facilitates electromagnetic coupling, exciting additional resonance points within the system. As observed in Figure 4, the Ant B curve exhibits flatter mid-band impedance matching alongside a new resonance at 3.55 GHz. This effectively broadens the high-frequency cutoff (VSWR < 2) from 3.4 GHz to 3.65 GHz, demonstrating the bandwidth-broadening capability of the proposed double-layer configuration.

In order to further broaden the operating frequency band, two structural extensions were carried out on the basis of Ant B, and the required bandwidth was achieved without changing the antenna size. The first optimization is the low-frequency extension. Adding downward vertical metal cylinders at the outermost vertices of the four radiating rings effectively lengthens the current transmission path on the antenna surface. This structural design aims to shift the resonance toward lower frequencies without increasing the horizontal size of the antenna. As shown by the curve of antenna C (Ant C) in Figure 3, after adding the metal cylinders, the lowest effective frequency point of the antenna shifts the lowest operating frequency from 2.06 GHz down to 1.98 GHz, broadening the operating bandwidth at the low-frequency end. To extend the high-frequency bandwidth, a second optimization was implemented by loading short-circuited metal stubs in the gaps near the central feeding region. These stubs regulate the current distribution to form a shorter path, allowing for precise fine-tuning of the impedance matching near 3.5 GHz and further improving the high-frequency bandwidth performance. It can be seen from Figure 4 that this optimization deepens the return loss pole in the range of 3.55 GHz to 3.6 GHz, improves the impedance bandwidth performance in the high-frequency band, and further extends the upper limit frequency to about 3.77 GHz. The finally evolved Ant C maintains VSWR < 2.0 over the ultra-wideband range of 1.98 GHz to 3.77 GHz, achieving the design goal of broadband impedance matching.

**Figure 5:**
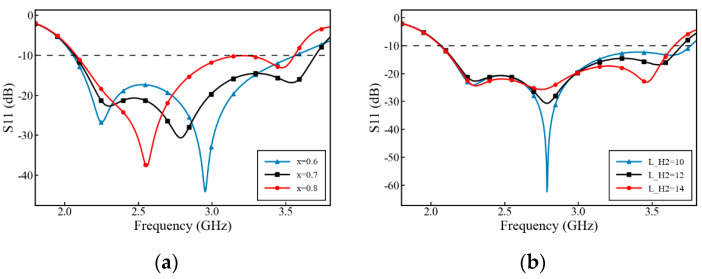
Simulated S11 of the low-frequency antenna for different parasitic structure parameters: (**a**) X; (**b**) L_H2.

To further investigate the specific influence of the parasitic structure parameters on the antenna impedance characteristics, a parameter scan was carried out on the scaling factor X of the parasitic element and the spacing L_H2 between the parasitic layer and the main radiating element, and the results are shown in Figure 5. As shown in Figure 5a, increasing the scaling factor X of the parasitic element also increases its equivalent electrical length. This causes the resonance point to shift significantly toward lower frequencies, rapidly degrading the matching above 3.1 GHz. When the size decreases, the in-band fluctuation is large and deterioration appears earlier at the high-frequency end. Therefore, setting X to 0.7 introduces a smooth resonance transition near 3.55 GHz. This maximizes the high-frequency impedance bandwidth while maintaining mid-band flatness. As shown in Figure 5b, the air spacing L_H2 primarily controls the vertical electromagnetic coupling between the parasitic layer and the bottom radiating element. When the spacing is small, the capacitive coupling between the two layers is too strong, which not only leads to an excessively deep resonance pole near 2.8 GHz, but also causes the impedance matching in the high-frequency band after 3.4 GHz to deteriorate earlier. Conversely, if the spacing is too large, the weakened coupling makes it difficult to effectively excite the parasitic element, thereby worsening the mid-band flatness. After comprehensive compromise and optimization, the air spacing is finally set to L_H2 = 12 mm in this design to ensure optimal return loss flatness and bandwidth coverage over the entire operating frequency band.

**Figure 6:**
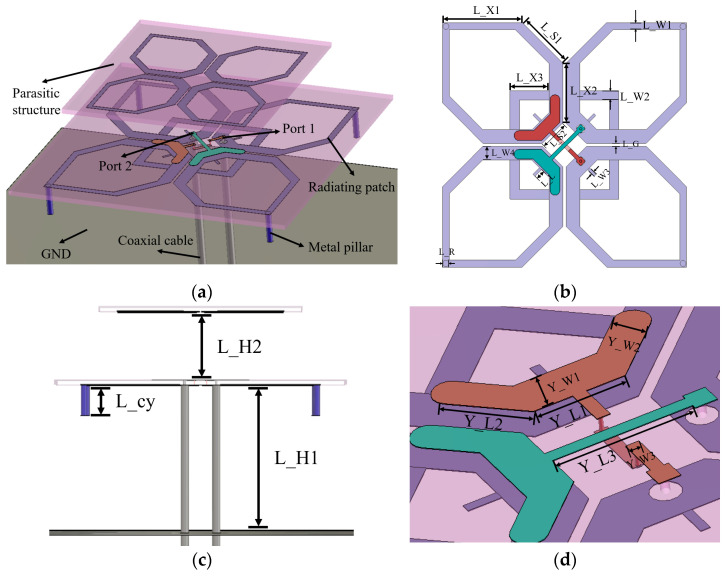
Structure and dimensions of the low-frequency antenna: (**a**) overall structure; (**b**) top view with detailed dimensional parameters; (**c**) side view; (**d**) Y-shaped feeding probe.

After parameter optimization using CST 2025 electromagnetic full-wave simulation software, the optimal physical dimensions of each structure of this antenna were finally determined. The overall structure of the low-frequency antenna is shown in Figure 6, and the specific parameters are shown in Table 2.

**Figure 7:**
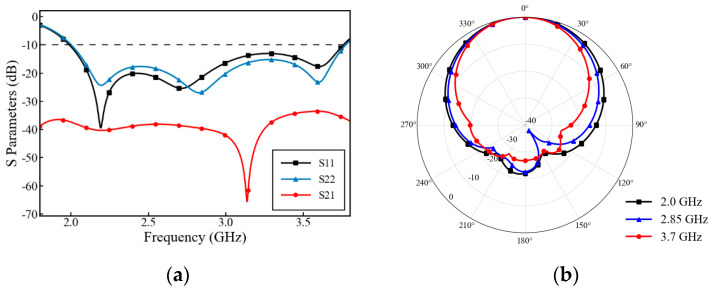
Simulated performance of the low-frequency antenna: (**a**) S-parameters; (**b**) normalized main-polarization radiation patterns at 2.0 GHz, 2.85 GHz, and 3.7 GHz.

Figure 7 shows the S-parameters and normalized radiation patterns of the antenna. Figure 7a clearly shows that within the wideband range of 1.98 GHz–3.77 GHz, the return loss for both ports exceeds 10 dB, and the port isolation is greater than 30 dB, meeting the commercial requirements of base station antennas. Figure 7b shows the normalized co-polarization radiation patterns at the lowest, center, and highest frequencies of the operating bandwidth. Within the entire ultra-wideband range, the antenna maintains highly stable directional radiation characteristics.

### 2.3. Shared-Aperture Antenna Array

**Figure 8:**
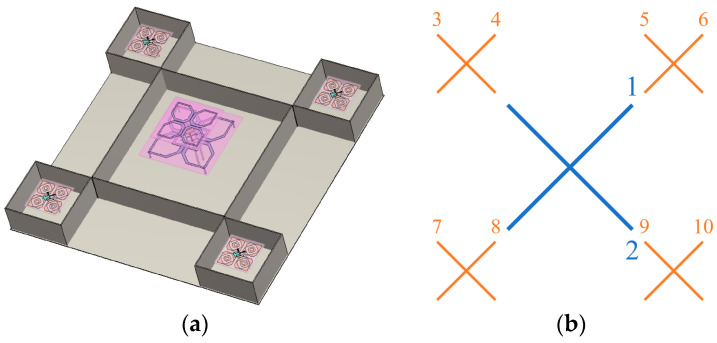
Overall layout and port configuration of the dual-band shared-aperture base station antenna: (**a**) 3D view of the antenna structure; (**b**) schematic diagram of the port numbering and feeding network.

Figure 8 shows the overall layout and port configuration of the dual-band shared-aperture base station antenna designed in this paper. In order to accommodate the low-frequency radiating patch with a larger size and reserve installation space for the metal partition, the spacing of each high-frequency antenna in the X-axis and Y-axis directions in Figure 8a is set to 130 mm. Figure 8b shows the excitation port configuration of the antenna. Ports 1 and 2 are the feeding ports of the low-frequency antenna, and ports 3 to 10 are the feeding ports of the high-frequency antennas.

In compact shared-aperture conditions, if no metal partition is loaded, there will be strong electromagnetic interference between the high-frequency and low-frequency antennas. This interference is mainly caused by the multiple resonance relationship between their operating wavelengths.

**Figure 9:**
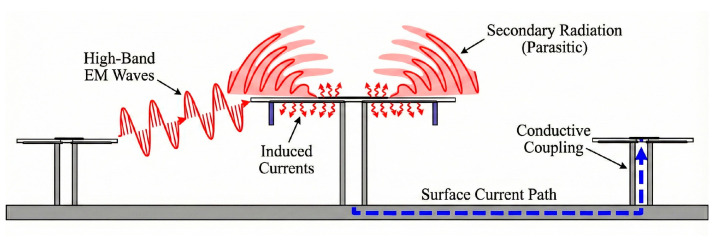
Illustration of electromagnetic interference mechanisms in the shared-aperture array.

When the high-frequency antenna operates, its spatial radiation wave directly irradiates the low-frequency antenna patch with a larger physical size and induces current, as shown by the red wave in Figure 9. This causes the low-frequency patch to become a parasitic secondary radiation source, and this part of the radiation field participates in the normal radiation of the high-frequency antenna, directly resulting in severe distortion of the radiation pattern in the high-frequency band. When the low-frequency antenna operates, the current flows along the ground plane to the coaxial line of the high-frequency antenna and then to the high-frequency radiating patch, as shown by the blue dashed arrows in Figure 8, thereby destroying the normal radiation of the low-frequency antenna. To solve the above electromagnetic interference problem, the overall antenna structure is finally designed in the form of loading metal partitions as shown in Figure 8a, aiming to reshape the boundary conditions, reduce the secondary radiation caused by the high-frequency antenna electromagnetic waves radiating onto the low-frequency antenna patch, and spatially block the propagation path of high-frequency electromagnetic waves.

**Figure 10:**
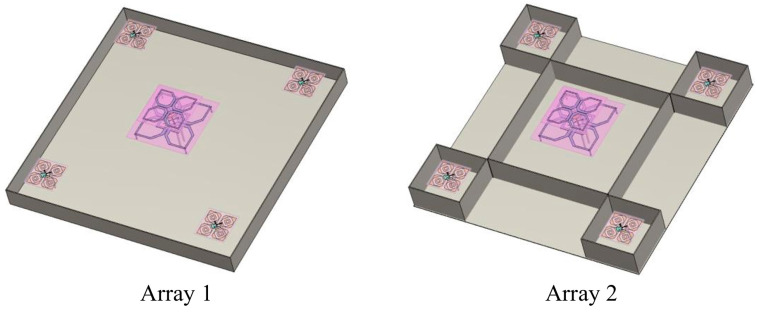
Two sets of reference frames for the antenna arrays.

For better illustration in the following, a reference group as shown in Figure 10 is established. Array 1 is the initial shared-aperture array without any isolation structure loaded, which is directly composed of one low-frequency radiating element and a group of 2 × 2 high-frequency antenna array. Array 2 adds a 20 mm high metal baffle on the basis of Array 1, enclosing each antenna to correct the beam-pointing distortion of the high-frequency antenna caused by interference.

**Figure 11:**
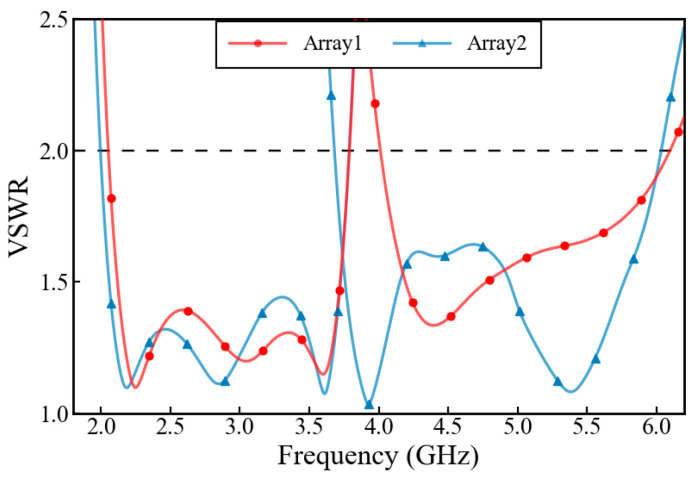
Comparison of the VSWR performance between Array1 (red) and Array2 (blue) from 2 GHz to 6 GHz.

Figure 11 shows the comparison curves of the VSWR of Array 1 and Array 2 between the 2–6 GHz frequency band. It can be seen from the red curve that due to the strong near-field coupling and spatial interference between antenna elements, the input impedance of the feeding port is affected by parasitic loading, resulting in failure to meet VSWR < 2 at the low-frequency region and at the 3.7 GHz frequency point. After inserting the metal baffle, the impedance matching characteristics of the array are significantly optimized. As shown by the blue curve in Figure 11, Array 2 perfectly achieves the design goal of VSWR < 2 within the required 2–3.77 GHz low-frequency operating band and 3.68–6 GHz high-frequency operating band of the system. The coupling between the baffle and the radiating patch as well as the coaxial probe generates additional distributed capacitance and distributed inductance. Through reasonable physical height design and spacing setting, this parasitic reactance network compensates for the inductive or capacitive mismatch of the antenna at the band edges, significantly broadening the impedance bandwidth of the dual bands.

**Figure 12:**
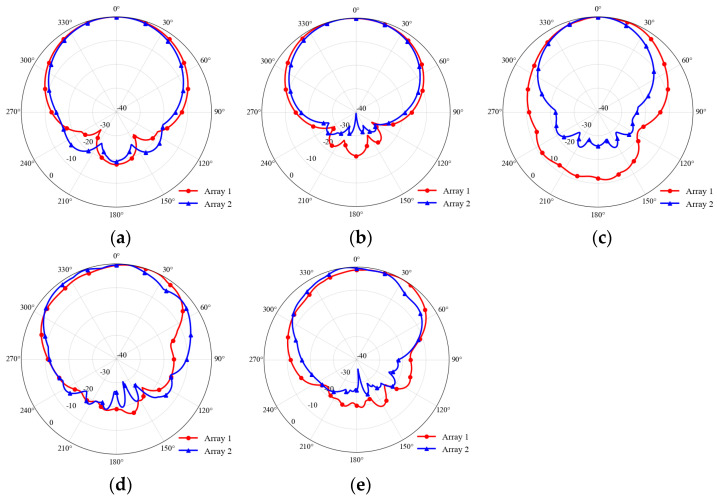
Normalized co-polarization radiation patterns of Array1 (red) and Array2 (blue) at different frequencies: (**a**) 2 GHz, (**b**) 2.85 GHz, (**c**) 3.7 GHz, (**d**) 5 GHz, (**e**) 6 GHz.

To verify the spatial radiation characteristics of the array over the entire operating bandwidth and the restoration effect of the baffle on the radiation pattern, Figure 12 selects typical frequency points in the high- and low-frequency bands and compares the normalized co-polarization radiation patterns of Array 1 (red curve) and Array 2 (blue curve). The three frequency points of 2 GHz, 2.85 GHz, and 3.7 GHz correspond to the lower edge, center frequency, and upper edge of the low-frequency operating band (2–3.77 GHz), respectively. Similarly, 3.68 GHz, 5 GHz, and 6 GHz correspond to the lower edge, center frequency, and upper-edge frequency points of the high-frequency antenna operating band (3.68–6 GHz). It can be seen from Figure 12a–c that after introducing the metal baffle, the radiation patterns at different frequency points are affected differently. At the 2 GHz frequency point (Figure 12a), the back lobe radiation of Array 2 (blue curve) after loading the baffle increases compared with the initial Array 1. The reason is that at lower frequencies, the wavelength is longer, and the metal baffle is electrically small at this time, causing edge diffraction of electromagnetic waves at the edge of the baffle. As shown by the 3.7 GHz result in Figure 12c, the baffle can effectively confine the radiated energy and push it toward the forward main lobe region, thereby significantly reducing the back lobe level. The beam correction effect of the baffle is particularly significant for antennas operating in the high-frequency band. As shown in Figure 12d,e, the radiation pattern of the initial structure (Array 1) exhibits severe asymmetric distortion. The main beam is significantly broadened, the maximum power direction is severely shifted, and strong parasitic side lobes appear. These issues are caused by secondary radiation interference, which occurs when high-frequency electromagnetic waves directly irradiate the low-frequency patch. After loading the metal baffle, Array 2 successfully blocks the direct radiation path of the high-frequency waves in physical space, reducing the interference of parasitic radiation sources. It can be clearly seen from the blue curve that the radiation pattern of the high-frequency antenna restores the original good symmetry and smoothness, and the main beam direction is perfectly corrected. The above results show that after adding the metal baffle, the structure can effectively correct the distortion of the radiation pattern.

## 3. Experimental Results and Analysis

**Figure 13:**
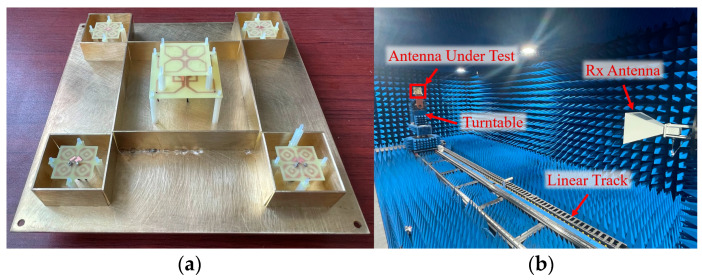
Fabricated prototype and measurement environment: (**a**) detailed view of the shared-aperture antenna assembly; (**b**) experimental setup in the microwave anechoic chamber.

In this section, the above dual-band shared-aperture base station antenna was fabricated and tested to verify the validity of the simulation results. The fabricated prototype of the antenna is shown in Figure 13. The high-frequency antenna dielectric substrate is fixed to the ground plane with nylon posts around its periphery. The low-frequency antenna is fixed to the ground plane at its four corners with nylon posts, and the parasitic structure is supported and fixed on the main radiating element with nylon posts. Finally, the metal partitions are inserted into the slots of the ground plane to complete the installation.

**Figure 14:**
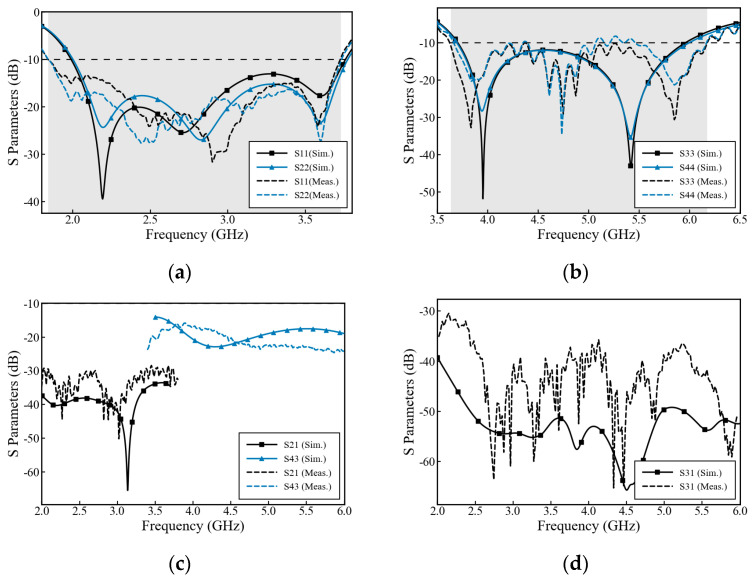
Comparison of simulated and measured S-parameters: (**a**) reflection coefficients of the low-band antenna, (**b**) reflection coefficients of the high-band antenna, (**c**) transmission coefficients of the high-band antenna and low-band antenna, (**d**) transmission coefficients between the high-band and low-band antennas. The solid lines denote simulation results, whereas the dashed lines denote measured results.

The network parameters of the antenna prototype were measured using a vector network analyzer (3674 L), in order to obtain the reflection coefficients of each port and the isolation between ports. Figure 14a shows that the simulated impedance bandwidth of the low-frequency antenna is 2–3.77 GHz (S11 < 10 dB), and the measured result is 1.84–3.72 GHz. Figure 14b shows that the simulated impedance bandwidth of ports 3 to 4 of the high-frequency antenna is 3.68–6 GHz (S11 < 10 dB), and the measured result is 3.63–6.3 GHz. The measured resonance frequency points and impedance bandwidth are highly consistent with the simulated results. It can be seen from Figure 14c,d that within the entire operating frequency band, the isolation is higher than 16 dB. The measured results ensure the independence and stability of the base station antenna during dual-band concurrent operation. As observed in Figure 14d, within the overlapping band (3.63–3.72 GHz), the measured S31 cross-band isolation is approximately 35 dB, which exhibits a noticeable around 15 dB degradation compared to the simulated value of nearly 50 dB. A certain degree of discrepancy exists between the simulated and measured results, which can be attributed to the following factors: While simulation models typically employ ideal waveguide ports for excitation, the physical prototype requires the installation of SubMiniature version A (SMA) coaxial connectors for feeding. During actual fabrication, the uneven application of solder, the non-ideal flatness of the soldered interfaces, and the inherent parasitic inductance and capacitance of the connectors introduce additional ohmic and impedance mismatch losses. Furthermore, slight electromagnetic leakage from these physical interfaces and testing cables degrades the cross-band isolation from the simulated 50 dB to the measured 35 dB. Notably, an isolation of 35 dB represents a mere 0.03% of coupled power (compared to 0.001% for 50 dB). This absolute leaked energy remains negligible and fully satisfies commercial base station requirements (typically >28 dB).

**Figure 15:**
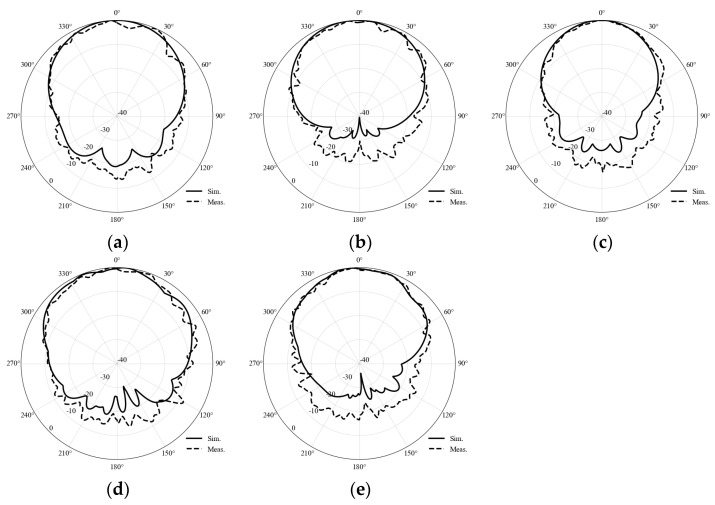
Comparison of simulated and measured radiation patterns at different frequencies: (**a**) 2 GHz, (**b**) 2.85 GHz, (**c**) 3.7 GHz, (**d**) 5 GHz, (**e**) 6 GHz.

The far-field radiation characteristics of the tested antenna were measured using a system mainly composed of three standard gain horn antennas at the transmitting end and the antenna under test at the receiving end. During the measurement, the antenna under test was firmly mounted on a low-dielectric-constant foam support and a high-precision three-dimensional turntable. In order to fully cover the broadband operating characteristics of the shared-aperture antenna, the radiation measurement process was sequentially conducted using the three horn antennas, which cover the frequency ranges of 1.72–2.61 GHz, 2.6–3.95 GHz, and 3.94–5.99 GHz, respectively. The radiation pattern measurement results are shown in Figure 15.

**Figure 16:**
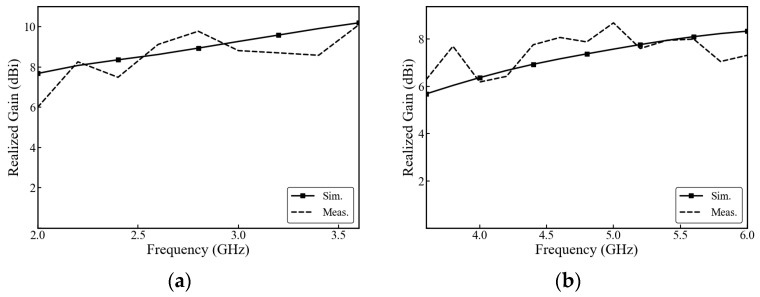
Comparison of simulated and measured realized gain: (**a**) low-band antenna, (**b**) high-band antenna. The solid lines denote simulation results, whereas the dashed lines denote measured results.

Figure 16 shows the gain curves of the dual-band shared-aperture antenna. In Figure 16a, the simulated gain of the low-frequency antenna is 7.8–10.2 dBi, and the measured result is 6–10.2 dBi. In Figure 16b, the simulated gain of the high-frequency antenna is 5.9–8.3 dBi, and the measured result is 6.1–8.5 dBi. The discrepancy between the simulated and measured gain at lower frequencies is primarily attributed to multipath interference within the practical measurement environment. Unlike the idealized full-wave simulation—which assumes perfect absorbing boundaries and the absence of physical support structures—the actual test environment introduces unintended scattering. At lower frequencies, the relatively large wavelength makes the antenna’s radiation highly susceptible to scattering from metallic mounting fixtures and positioners inside the anechoic chamber. Additionally, potential common-mode currents on the outer conductor of the coaxial feed cable can act as secondary radiating sources. The superposition of these scattered waves with the main radiation leads to constructive interference at certain frequencies (e.g., reaching approximately 9.8 dBi near 2.8 GHz) and destructive interference at others (e.g., dropping to 6.0 dBi at 2.0 GHz), thereby producing the observed oscillatory behavior in the measured realized gain.

To objectively evaluate the overall goodness of the proposed design against similar antennas in the literature, it is essential to consider the comprehensive trade-off between multi-band performance (e.g., bandwidth) and structural modifications (e.g., decoupling complexity). This multi-objective trade-off evaluation methodology is highly emphasized in the design of advanced future wireless systems [15]. As summarized in Table 3, we evaluate this performance-decoupling trade-off by comparing the fractional bandwidths (FBW) against various cross-band decoupling strategies. In shared-aperture arrays, achieving effective decoupling often comes at the severe expense of increased structural complexity—such as utilizing dielectric-loaded transparent elements [8], inhomogeneous metasurfaces [12], or intricate 3-D chokes [13]. By adopting this trade-off perspective, the advantages of the proposed design become highly evident. Instead of employing highly complex structures, the strategic utilization of simple, integrally formed physical metal baffles avoids the extreme complexity penalty. Consequently, the proposed antenna strikes an optimal structural-performance trade-off: it relies on minimal structural modifications while successfully securing ultra-wide fractional bandwidths (achieving 67.6% and 53.8%) and maintaining robust cross-band isolation.

## 4. Conclusions

This paper presents a non-overlapping planar cross-arranged ultra-wideband shared-aperture base station antenna array targeting the 2–6 GHz application band, aimed at addressing the challenges of multi-band integration and electromagnetic coupling interference in limited 5G deployment spaces. To meet these challenges, the low- and high-frequency modules are independently and cooperatively designed: the low-frequency module (1.84–3.72 GHz) employs a double-layer parasitic coupling structure to expand the impedance bandwidth, while the high-frequency module (3.63–6.3 GHz) uses chamfered slotted patches to achieve ultra-wideband coverage. To suppress high-frequency radiation pattern distortion caused by strong near-field coupling and secondary radiation in the shared-aperture layout, metal baffles are innovatively introduced around the antenna elements. These baffles reshape the boundary conditions and physically block electromagnetic coupling paths, significantly mitigating interference. Both simulation and measurement results show close agreement, with a VSWR below 2 across the entire operating band and port isolation exceeding 35 dB within the overlapping 3.63–3.72 GHz band. In addition, the metal baffles not only improve impedance matching at the band edges but also successfully correct high-frequency pattern distortion, ensuring highly stable directional radiation across the full frequency range. The maximum gain of the low-frequency antenna is 6–10.2 dBi, while the high-frequency antenna achieves 6.1–8.5 dBi. Overall, this design demonstrates excellent wideband impedance matching, high isolation, and stable radiation performance, providing an efficient, robust, and manufacturable solution for next-generation highly integrated multi-band base station antennas.

## Figures and Tables

**Table 1:** List of parameter values for the high-frequency antenna.

Parameter	Value (mm)	Parameter	Value (mm)	Parameter	Value (mm)
H_X1	7.5	H_S1	2.1	H_G1	1.7
H_X2	6.1	H_S2	4.55	H_TL	3.55
H_X3	1.6	H_S3	2.55	H_TW	1.3
H_X4	0.9	H_S4	1.25	H_H	15.9

**Table 2:** List of parameter values for the low-frequency antenna.

Parameter	Value (mm)	Parameter	Value (mm)	Parameter	Value (mm)
L_X1	12.8	L_W3	0.4	L_cy	5.0
L_X2	9.5	L_W4	2.2	Y_L1	4.1
L_X3	6.2	L_G	0.5	Y_L2	3.65
L_S1	9.15	L_L	1.5	Y_L3	6.3
L_S2	4.6	L_R	1.0	Y_W1	2.0
L_W1	0.9	L_H1	24.2	Y_W2	1.55
L_W2	1.5	L_H2	12.0	Y_W3	0.6

**Table 3:** Comparison of the proposed antenna with similar antennas in the literature.

Ref.	LB Operating Band (GHz)/FBW (%)	HB Operating Band (GHz)/FBW (%)	Cross-Band Decoupling Strategy	Array Configuration
[8]	0.76–1.03 (30.0%)	3.4–5.0 (38.0%)	Dielectric Loaded Transparent	1 LB + 16 HB
[10]	0.69–0.96 (32.7%)	1.71–2.69 (44.5%)	EM-Transparent Element	1 LB + 2 HB
[12]	0.57–0.97 (51.9%)	1.51–2.95 (64.5%)	Inhomogeneous Metasurface	1 LB + 2 HB
[13]	0.68–0.98 (36.1%)	1.7–2.7 (45.4%)	3-D Chokes & Path Extension	1 LB + 4 HB
This Work	1.84–3.72 (67.6%)	3.63–6.30 (53.8%)	Metal Baffles	1 LB + 4 HB

## Data Availability

The original contributions presented in this study are included in the article. Further inquiries can be directed to the corresponding author.

## Abbreviations

The following abbreviations are used in this manuscript:

VSWR	Voltage Standing Wave Ratio
3GPP	3rd Generation Partnership Project
RF	Radio Frequency
FDD	Frequency Division Duplex
TDD	Time Division Duplex
LTE	Long Term Evolution
NR	New Radio
CBRS	Citizens Broadband Radio Service
LAA	Licensed Assisted Access
FSS	Frequency Selective Surface
LC	Inductor–Capacitor
SMA	SubMiniature version A
